# Calcified Chondroid Mesenchymal Neoplasm of the Masticator Space: A Case Report

**DOI:** 10.7759/cureus.85068

**Published:** 2025-05-30

**Authors:** Yuan-Xian Too, Chen-Yu Hsiao, Tai-Jui Chen

**Affiliations:** 1 Department of Diagnostic Radiology, Shin Kong Wu Ho-Su Memorial Hospital, Taipei, TWN

**Keywords:** calcified chondroid mesenchymal neoplasm, fn1 fusion, masticator space tumor, mesenchymal tumor, soft tissue tumor

## Abstract

Calcified chondroid mesenchymal neoplasm (CCMN) is a rare, recently defined tumor characterized by mesenchymal tissue with chondroid features and calcification. It typically harbors an FN1 fusion and shows a predilection for the temporomandibular joint and distal extremities. CCMN is benign and has not been associated with malignant behavior. We present the case of a 61-year-old woman with an eight-year history of a progressively enlarging, palpable mass in the left parotid region, associated with ipsilateral hearing loss and facial palsy. Imaging studies, including computed tomography (CT) and magnetic resonance imaging (MRI), revealed a well-defined tumor located between the left masseter muscle and parotid gland, extending into the left supratemporal fossa. The lesion exhibited heterogeneous soft tissue density with irregular intratumoral calcifications and moderate contrast enhancement. Ultrasound demonstrated a hypo- to isoechoic nodular lesion within the left parotid gland. Histopathological evaluation, in correlation with imaging findings, confirmed the diagnosis of CCMN. Surgical excision was performed, and postoperative imaging showed no residual tumor. This case highlights the importance of considering CCMN in the differential diagnosis of calcified soft tissue tumors in the head and neck region.

## Introduction

Calcified chondroid mesenchymal neoplasm (CCMN) is a rare and relatively newly recognized entity in the field of soft tissue tumors. Described for the first time by Liu et al. in 2021, CCMNs are characterized by a combination of mesenchymal tissue with chondroid (cartilage-like) features and areas of calcification [1]. While these tumors have been most commonly identified in the temporomandibular joint region and distal extremities, their clinical and radiological presentation can vary, making diagnosis a challenge [2]. Histologically, CCMNs often present ovoid to spindled cells with a cartilaginous matrix, and they can exhibit irregular, coarse calcifications within the soft tissues [3]. A study analyzing 33 cases of CCMN reported a nearly equal gender distribution, with 17 males and 16 females, and a mean age of 51.3 years [4]. This finding suggests that CCMN does not exhibit a strong gender predilection, affecting both males and females across a wide age range.

The diagnosis of CCMN is typically supported by imaging modalities, including computed tomography (CT), magnetic resonance imaging (MRI), and ultrasound, which reveal a well-defined mass with heterogeneous characteristics and distinct calcific components. In addition, recent studies have identified the presence of gene fusions, notably involving FN1 and receptor tyrosine kinases, which may provide a molecular basis for these tumors [1].

Despite being benign, enlarged CCMNs can present with clinical symptoms, often due to mass effect or location, such as in the case of the temporomandibular joint or parotid gland regions. Our case report highlights the imaging findings and clinical presentation of CCMN in a 61-year-old woman, underscoring the importance of recognizing this rare tumor and its characteristic features in radiological studies.

## Case presentation

In June 2024, a 61-year-old woman presented to the outpatient clinic with a long-standing history of a progressively enlarging left parotid mass, which she had noticed over the past eight years. The mass had gradually increased in size, though it was not initially painful. In addition to the mass, the patient reported left-sided hearing loss and facial palsy, which had developed over the past two years. Besides, she also suffered from left diplopia, left blurred vision, and left wisdom tooth pain in the past few months. She denied any history of trauma, fever, or weight loss. The patient's medical history was notable for hypertension, which was well-controlled with medication. There was no significant history of previous malignancy or surgical interventions in the head and neck region.

On physical examination, the patient appeared well-nourished and in no acute distress. Inspection of the left parotid region revealed a firm, non-tender, mobile mass. Palpation confirmed a well-defined, firm mass, approximately 4 cm in diameter, located in the left preauricular region. A neurological examination showed facial weakness on the left side, indicating possible facial palsy.

Pure-tone audiometry revealed bilateral sensorineural hearing loss ranging from normal to moderate severity, which was considered age-related and not directly attributable to the parotid mass. Ophthalmologic examination showed mild left-sided blepharitis, without evidence of cranial nerve palsy, ophthalmoplegia, or other abnormalities suggestive of direct orbital or neurological involvement. The reported diplopia and blurred vision were intermittent and likely unrelated to the primary pathology.

Given the chronicity of the symptoms and the size of the mass, imaging studies were ordered to assess the extent of the lesion and its relationship to surrounding structures.

Imaging findings

**Figure 1:**
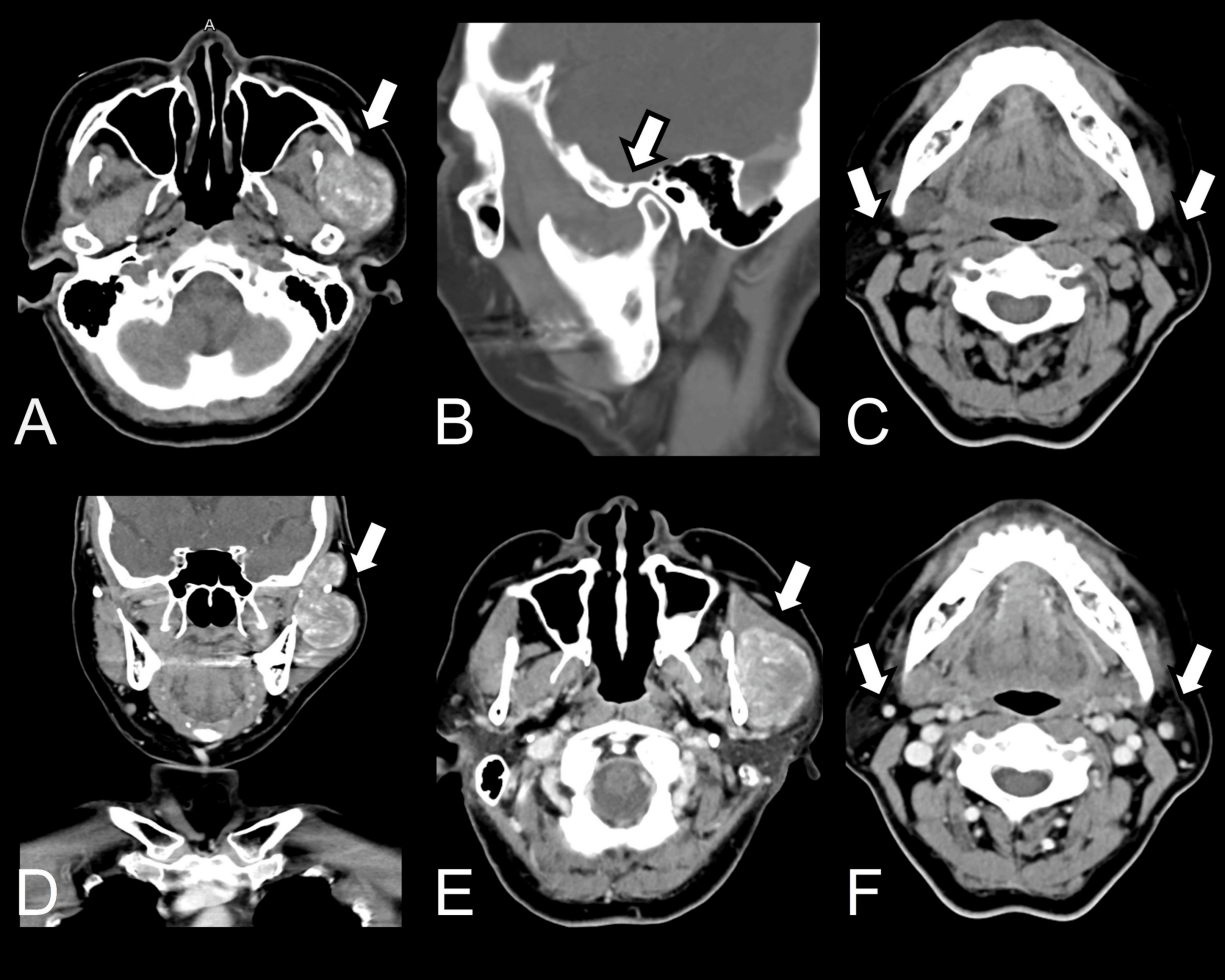
CT scan. (A) Axial view demonstrates a soft tissue mass encasing the left masseteric region and exhibiting internal calcifications. (B) Sagittal view shows no definite erosion of the overlying temporal and mandibular bone. (C and F) Images show non-specific lymph nodes at level IIa. (D and E) Post-contrast images show heterogeneous enhancement. CT: computed tomography

A contrast-enhanced CT scan (Figure 1) of the head and neck was performed, which revealed a well-defined, heterogeneously enhancing mass located between the left masseter muscle and the left parotid gland, with extension into the left supratemporal fossa. The tumor measured approximately 3.25 × 4.34 × 6.1 cm, causing peripheral muscle swelling. The mass showed irregular intratumoral calcifications, which were scattered, coarse, and grungy in appearance. These findings were suggestive of a soft tissue neoplasm with calcified components. The mass had a moderate enhancement on post-contrast images, and no evidence of bony involvement was noted. 

**Figure 2:**
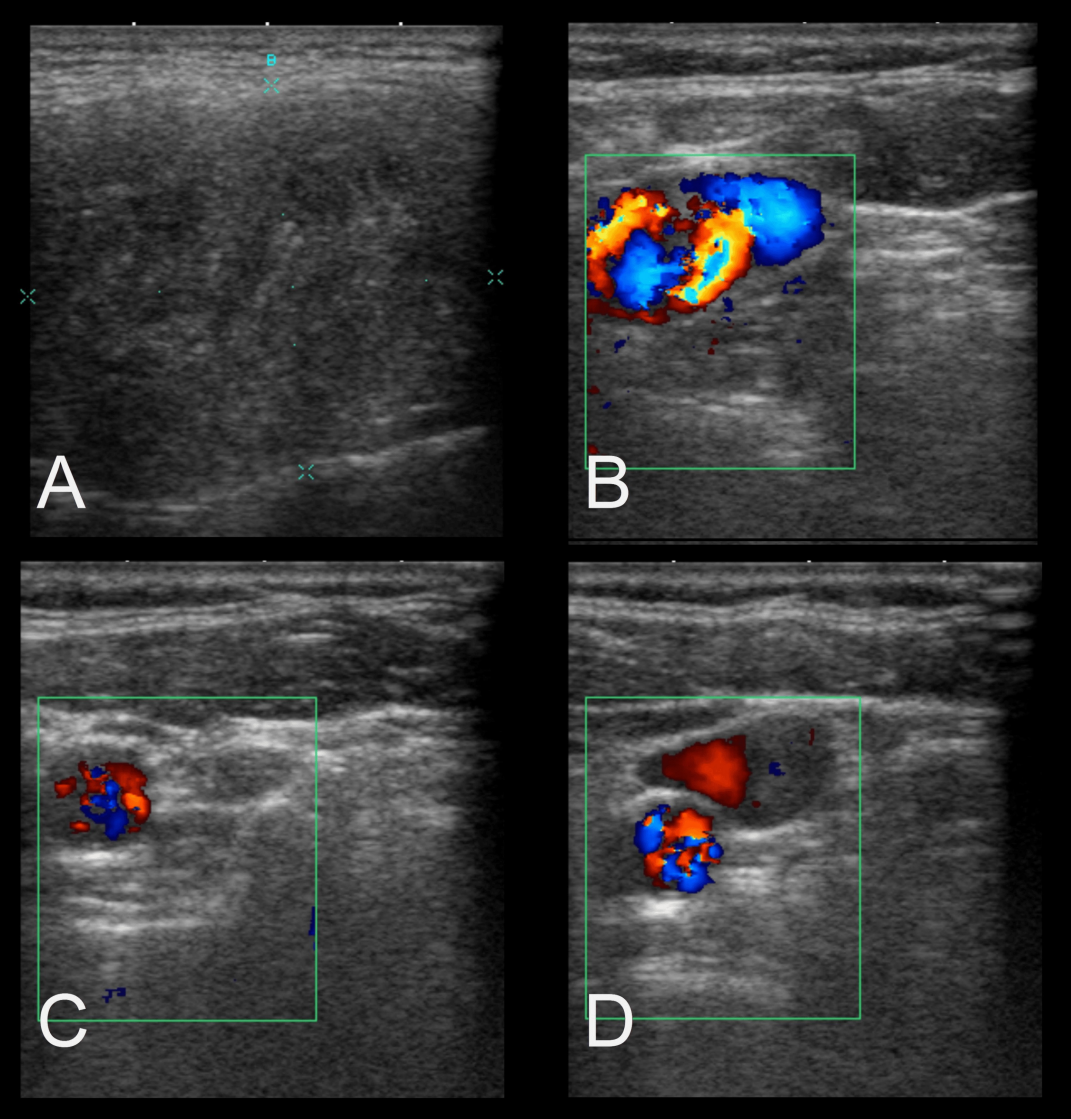
Neck ultrasound. (A) One hypo- to isoechoic nodular lesion at the left parotid gland, about 3.51 × 2.97 cm in size. (B, C, and D) The nodule demonstrates heterogeneous internal echotexture.

Ultrasound examination (Figure 2) was also conducted to evaluate the parotid region further. The ultrasound revealed a hypo- to isoechoic nodular lesion within the left parotid gland, measuring 3.51 × 2.97 cm. The lesion was well-circumscribed but showed heterogeneous internal echotexture with areas of calcification, correlating with the CT findings. There was no evidence of lymphadenopathy or fluid collection, further suggesting a benign lesion.

To further evaluate the lesion and obtain tissue for pathological evaluation, fine needle aspiration cytology (FNAC) was performed. The FNAC results were negative for malignancy, with no evidence of atypical or malignant cells. The cytological findings were suggestive of a benign mesenchymal neoplasm, but they did not provide a definitive diagnosis.

**Figure 3:**
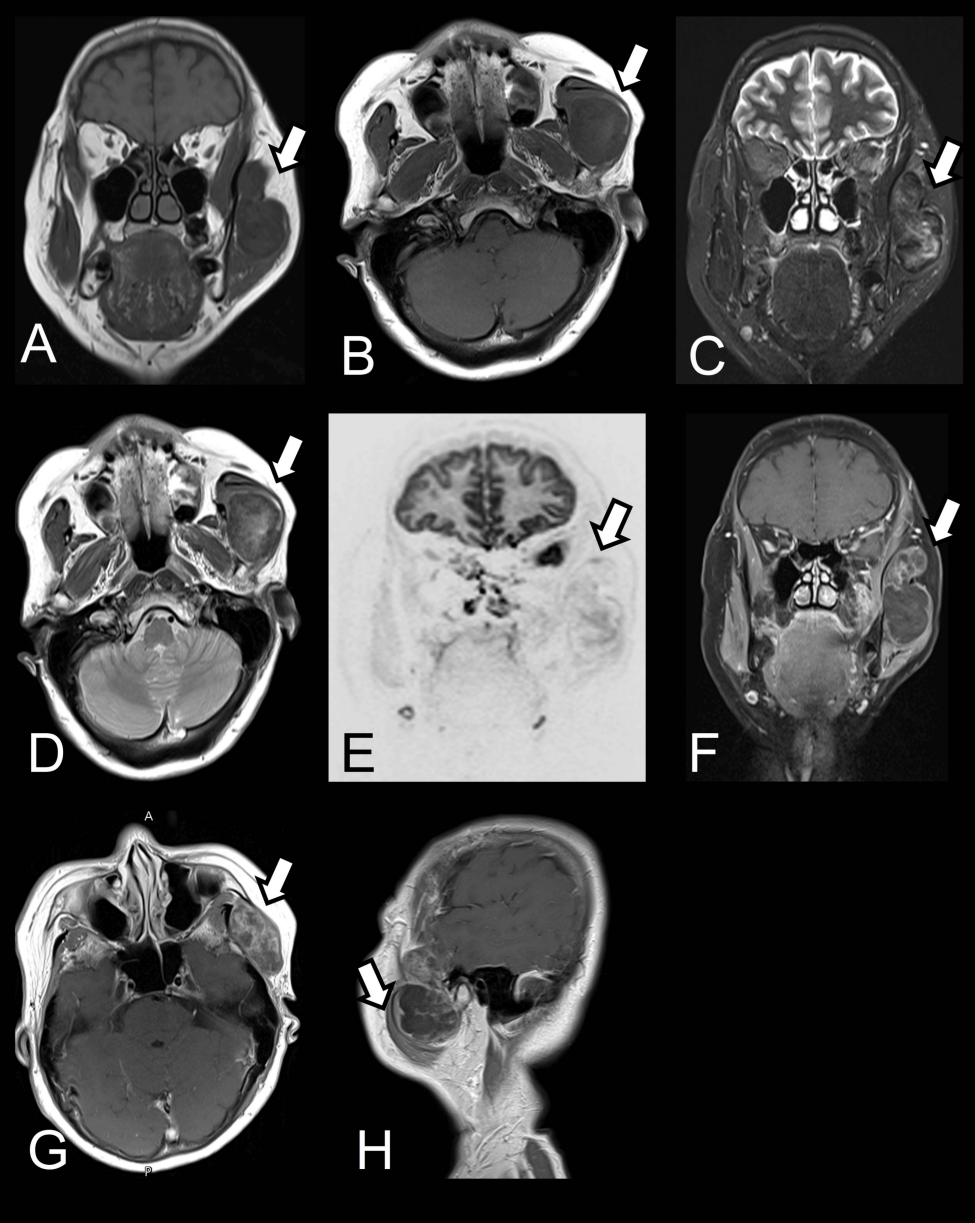
MRI. (A and B) T1-weighted images demonstrate a low signal, capsular enhanced mass around the left masticator space with the involvement of the left supratemporal fossa. (C) T2-weighted, (D) proton density, and (E) diffusion-weighted images show intralesion moderate signal without peripheral edema. (F, G, and H) Post-contrast images show heterogeneous enhancement. MRI: magnetic resonance imaging

Given the imaging features and clinical presentation, the lesion was suspected to be a soft tissue neoplasm, and additional imaging with MRI was requested for better characterization. The MRI of the head and neck (Figure 3) showed a mass with a low signal intensity on T1-weighted image (T1WI) and an increased signal on T2-weighted image (T2WI) with calcifications and cartilaginous components. There are some foci areas that show relative hyperintensity on T1WI and T2WI and demonstrated minimal increasing fluid signal on T2W, proton density (PD), and diffusion-weighted images (DWI). Post-contrast imaging revealed significant heterogeneous enhancement, especially at the upper tumor region, further supporting its neoplastic nature. The tumor extended into the supratemporal fossa, raising concerns about potential involvement with adjacent structures, including the facial nerve. However, MRI findings did not show any definite hyperintensity of the left facial nerve on T2WI.

Nerve conduction studies

To further assess potential nerve involvement, nerve conduction studies were conducted, revealing left facial neuropathy, predominantly affecting the temporal branch, with partial reinnervation in the zygomatic branch. The compound muscle action potential (CMAP) amplitude was reduced by 15% compared to the normal right side.

Diagnosis and surgical intervention

Based on the imaging findings and clinical presentation, the patient received an operation for tumor excision in July 2024. Under general anesthesia, a preauricular incision with a cervical extension was made to expose the parotid region. Dissection proceeded to separate the parotid gland from surrounding structures, including the sternocleidomastoid muscle and external auditory canal. The facial nerve was identified and preserved. The tumor, located between the parotid's superficial lobe and masseter muscle, was entrapped in the zygomatic arch. Osteotomy of the zygomatic arch was performed to release the tumor, which was then exteriorized. A frozen section confirmed the lesion is benign. The arch was repositioned with fixation, and the incision was closed, with the patient tolerating the procedure well.

Upon surgical excision of the mass, a frozen section was performed. The specimen measured 6.5 × 4 × 3 cm and weighed 43 grams. Gross examination revealed a well-circumscribed, multinodular tumor with firm consistency and calcified chondroid tissue, indicating the tumor was confined to surrounding soft tissues. Microscopic examination showed a hypocellular, lobulated tumor with mildly atypical cells in a cartilaginous and calcified stroma, along with multinucleated giant cells and hemosiderin pigment. Histopathologic examination confirmed the diagnosis of CCMN.

Postoperative course and follow-up

**Figure 4:**
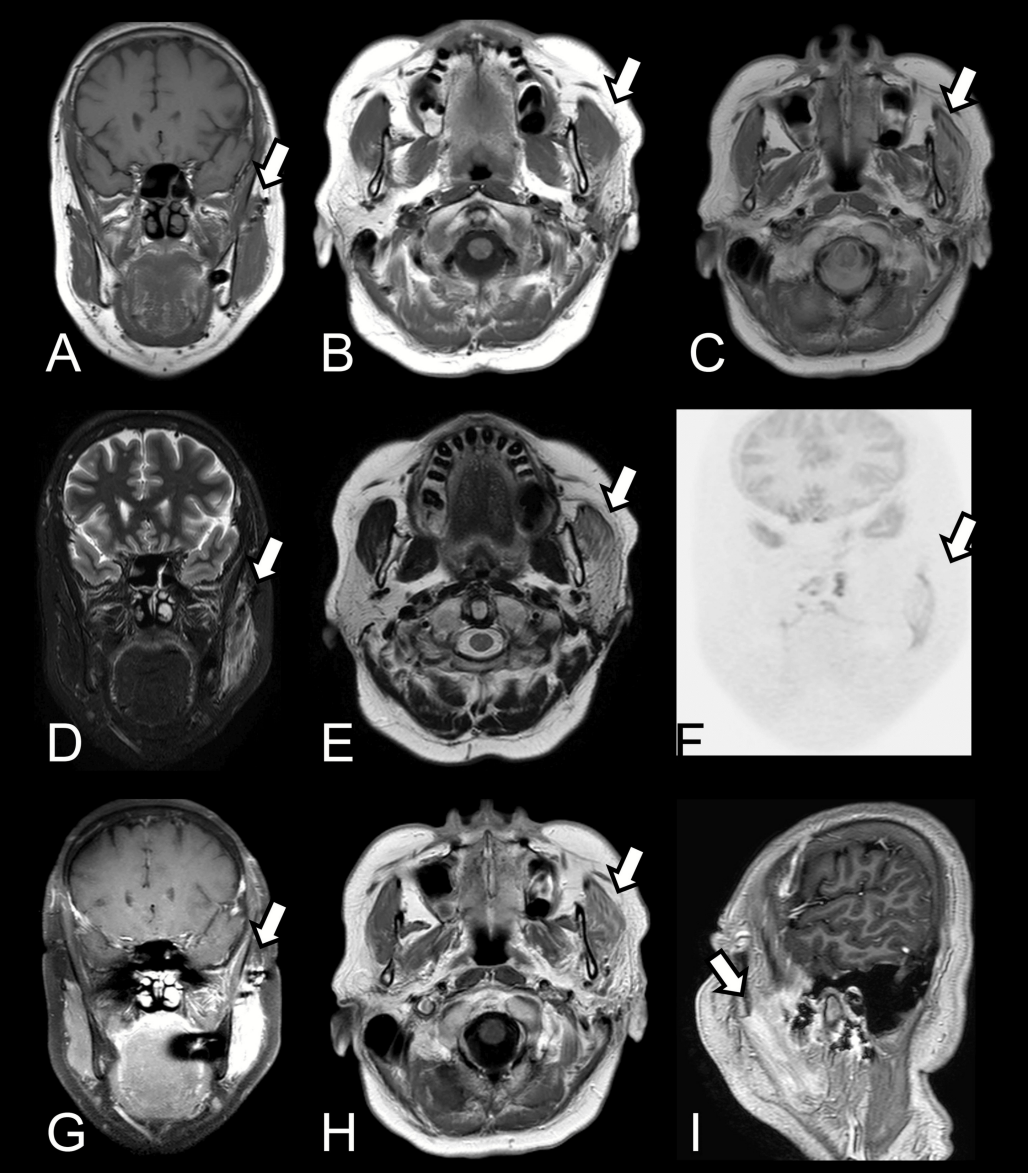
MRI at the three-month postoperative follow-up. (A and B) T1-weighted, (C) proton density, (D) STIR, (E) T2-weighted, (F) diffusion-weighted, and (G, H, and I) post-contrast images indicate status post operation without definite residual tumor in between the left masseter muscle and left parotid gland. Hyperintense T2 signal change in the left masseter muscle, probably due to postoperative inflammatory process. MRI: magnetic resonance imaging; STIR: short tau inversion recovery

**Figure 5:**
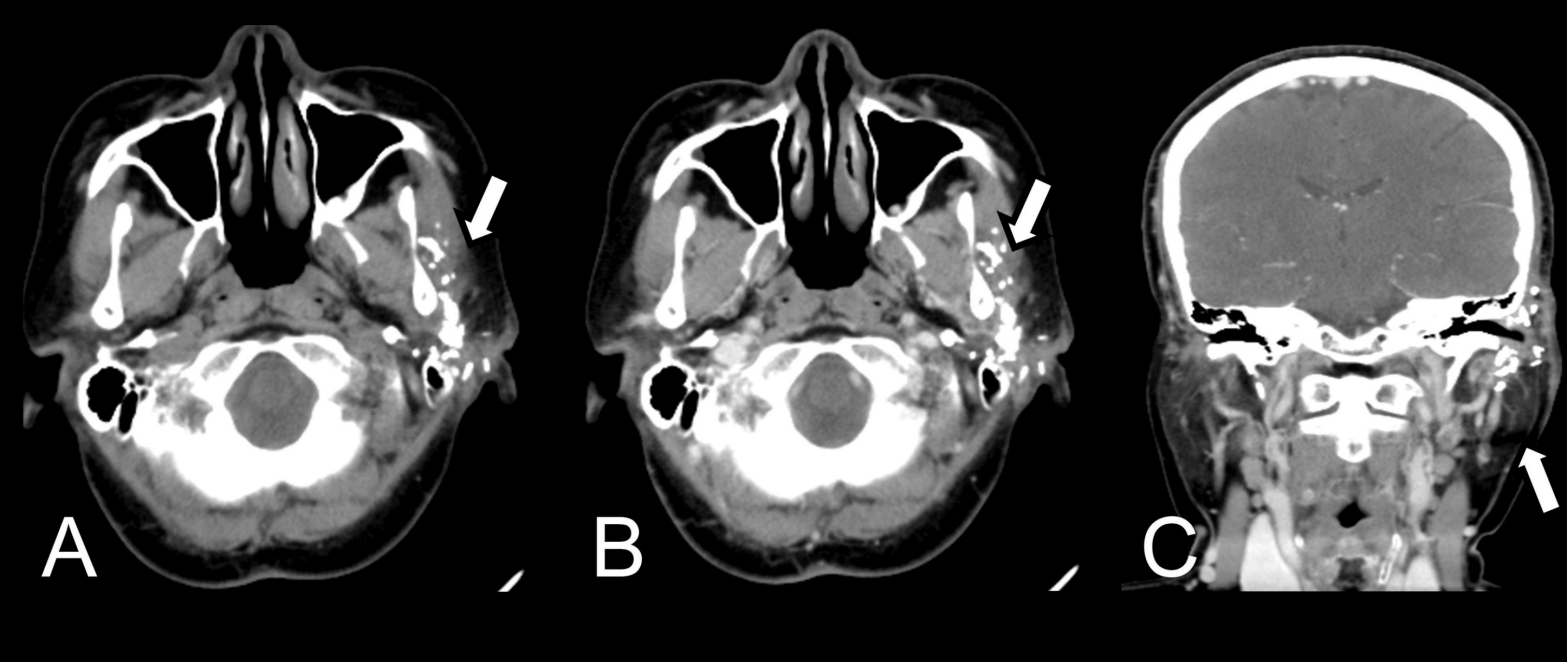
CT scan at the six-month postoperative follow-up. (A) Non-contrast and (B and C) post-contrast images show no obvious space-taking tumor identified. Focal skin thickening probably scar tissue and retained surgical materials. CT: computed tomography

The patient's postoperative course was uncomplicated. She had a well-healing surgical wound and continuously received a series of electrotherapy for her left facial nerve function. Postoperative imaging, including CT (Figure 4) and MRI (Figure 5), was performed postoperative three and six months, respectively, to assess for any residual tumor. Both imaging modalities confirmed the complete excision of the mass with no evidence of recurrence. At the six-month follow-up, the patient remained symptom-free, with no recurrence of the mass or facial palsy. Her facial nerve function had improved to baseline, and there were no further neurological deficits.

## Discussion

We presented the case of a 61-year-old woman with a progressively palpable left parotid mass for eight years, accompanied by left hearing loss and left diplopia. However, both symptoms were evaluated and found to be unrelated to the mass. CT imaging revealed a soft tissue mass with amorphous calcifications located between the left masseter muscle and parotid gland, without evidence of regional bone erosion. Ultrasound showed a hypo- to isoechoic nodular lesion. MRI revealed a soft tissue mass with low signal intensity on T1WI and scattered calcifications, intralesional foci of high signal intensity on T2-weighted sequences, and no obvious perilesional edema on fluid-sensitive sequences, and heterogeneous enhancement was noted. These findings suggest an encapsulated soft tissue component, a slow-growing, benign, but complex lesion, which is a highly valuable case for in-depth discussion.

CCMN is a recently defined category of tumors characterized by chondroid differentiation and calcification, often associated with specific genetic alterations, particularly FN1-receptor tyrosine kinase fusions [4,5]. These tumors typically present as painful soft tissue masses, commonly located in the extremities and around temporomandibular joints, consistent with our case [4]. CCMN can be misdiagnosed as benign conditions such as calcific tendinitis due to overlapping clinical features [4]. Histologically, CCMNs exhibit a lobular architecture with irregular, lace-like calcifications and polygonal to stellate tumor cells within a chondroid matrix [1]. The typical radiological appearance of CCMNs includes a well-defined mass with heterogeneous soft tissue composition and varying degrees of calcification. These tumors generally have slow, progressive growth and may cause mass effects on adjacent structures, depending on their location. Despite their unusual appearance, CCMNs are benign, with no documented cases of malignant transformation or metastasis after complete excision [4]. Surgical excision is the treatment of choice, and prognosis following complete excision is excellent, with no recurrence or long-term complications [2]. 

The rarity of CCMNs and their overlapping features with other soft tissue tumors, such as chondromas, chordomas, chondrosarcomas, and osteosarcomas, make them a diagnostic challenge, especially when dealing with lesions in the head and neck region [6-8]. To aid in accurate diagnosis, it is important to understand the key distinguishing features that differentiate CCMNs from these similar entities. The following points highlight the key points used to differentiate CCMNs from chondromas, chordomas, chondrosarcomas, and osteosarcomas.

Chondromas are benign soft tissue tumors of hyaline or myxoid cartilage originating in extraosseous and extrasynovial locations [9-11]. Unlike CCMNs, chondromas are generally located within the fingers on hands, toes, and feet and uncommonly in the trunk or head and neck [9]. Rarely, there is a case review for a right parotid chondroma, which has multiple small punctate and popcorn-like calcifications that are consistent with a chondroid matrix [12,13]. Radiologically, soft tissue chondromas typically appear well-defined and heterogeneous, showing hypointense to isointense signals on T1WI and hyperintense signals on T2WI [11,12]. Furthermore, even though some soft tissue chondromas are associated with FN1 gene rearrangements [10], they rarely can have moderate pleomorphism and typically do not exhibit hypercellular areas with cells resembling chondroblasts, which are characteristic of calcified chondroid mesenchymal tumor [1].

Chordomas arise from the remnants of the notochord, typically occurring in the sacrococcygeal region or the clivus at the skull base [14-17]. Intracranial chordomas are usually centrally located and well-circumscribed and tend to have destructive lytic lesions, sometimes with marginal sclerosis [18-21]. MRI showed most T2WI exhibits a very high signal, which was not seen in our patient's CCMN. Histopathologically, chordomas are characterized by the presence of fluid and gelatinous mucoid substances and necrotic areas that are absent in CCMNs. CCMNs, on the other hand, consist of a mesenchymal tissue matrix with cartilaginous differentiation and calcification without lytic, destructive behavior typical of chordomas. Clinically, metastatic spread of chordomas is observed in 7-14% of patients and includes nodal, pulmonary, bone, cerebral, or abdominal visceral involvement, predominantly from massive tumors [8,16], whereas CCMNs are typically asymptomatic or cause mild symptoms due to their slow growth.

Chondrosarcoma, a malignant cartilage-producing tumor, can show similar radiological features to CCMN, such as the presence of calcification, and is found in older patients [8,17,18]. However, chondrosarcomas are typically more invasive and destructive, often showing bone involvement, cortical breaches, and soft tissue extension. These aggressive characteristics were not present in our case, where the CCMN was confined to soft tissues and demonstrated a well-circumscribed border. Chondrosarcomas are also histologically more cellular, with increased atypia, pleomorphism, and mitotic activity, which distinguishes them from the benign, well-differentiated nature of CCMNs [17,18]. On MRI, chondrosarcomas tend to demonstrate a heterogeneous signal intensity with regions of low signal on T1WI and obviously high signal on T2WI, due to the cartilage being a hydrophilic tissue with high water content [19], a feature that differs from the findings in CCMNs.

Osteosarcoma, a highly aggressive bone tumor, can occasionally present in the head and neck, typically involving the jaw or paranasal sinuses [20,21]. It is characterized by mixed lytic and sclerotic areas, periosteal reactions such as the sunburst pattern, and cortical bone destruction [22]. The slow-growing, non-invasive nature of the CCMNs, along with their well-defined and encapsulated borders and absence of aggressive features, helped distinguish it from osteosarcoma. In MRI, the classic appearance of osteosarcoma is a mass with inhomogeneous low signal intensity on T1WI, high signal intensity on T2WI, and significant enhancement after contrast injection [8]. Due to their aggressive features, they often have peritumoral edema, seen with high signal intensity on T2WI [23,24]. The presence of calcification in CCMNs might initially raise concern for osteosarcoma, but the benign and non-destructive nature of CCMNs provides the key differentiating factor.

Key histologic features of CCMNs include the presence of ovoid to spindled cells within a chondroid to grungy matrix displaying lobular growth with occasional osteoclast-like giant cells [3]. Recent molecular studies of CCMNs have revealed the presence of FN1 gene fusions, involving fibronectin-1 and receptor tyrosine kinases such as FGFR1, FGFR2, or TEK [1,25]. These genetic alterations likely contribute to the formation of the mesenchymal tissue matrix and cartilaginous differentiation that characterize CCMNs. The FN1 gene fusions make them a unique feature of CCMNs and help to differentiate them from tumors such as chondrosarcoma and osteosarcoma, which lack these specific genetic alterations [3]. Though molecular studies were not performed in this case, the characteristic radiological and histopathological features, coupled with the patient's benign clinical course, strongly supported the diagnosis of CCMN.

Clinical management and prognosis

CCMN is a benign tumor, and the primary treatment is complete surgical excision. The patient in this case underwent successful resection of the tumor, with postoperative imaging showing no residual mass. As CCMNs are benign, the prognosis following complete surgical excision is excellent, with no reported cases of recurrence or metastasis in the literature [4,24]. However, because CCMNs are rare and have a potential for slow growth, regular follow-up imaging is recommended to monitor for any recurrence, although the risk of recurrence is low. The absence of malignant features and the tumor's well-defined nature support the conclusion that complete excision provides excellent long-term outcomes.

## Conclusions

CCMN is a rare, benign soft tissue tumor that can mimic other, more common tumors such as chondromas, chordomas, chondrosarcomas, and osteosarcomas due to overlapping radiologic features. However, careful radiological evaluation, histopathological examination, and consideration of the molecular genetic characteristics can help differentiate CCMN from these other entities. The indolent nature of CCMNs and the excellent prognosis following surgical excision emphasize the importance of an accurate diagnosis and appropriate management, avoiding unnecessary aggressive treatments typically reserved for malignant tumors.
